# Studies on immune response to Newcastle disease virus in broiler chickens fed with *Lactobacillus reuteri* PIA16 isolated from the gut of indigenous chicken of Assam, India

**DOI:** 10.14202/vetworld.2019.1251-1255

**Published:** 2019-08-15

**Authors:** Gaichamdinliu Gonmei, Deben Sapcota, Girin Kumar Saikia, Pankaj Deka, Joga Dev Mahanta, Niranjan Kalita, Bibeka Nanda Saikia, Jitendra Kumar Talukdar

**Affiliations:** 1 Department of Poultry Science, College of Veterinary Science, Assam Agricultural University, Assam, India; 2 Department of Animal Microbiology, College of Veterinary Science, Assam Agricultural University, Assam, India; 3 Department of Animal Nutrition, College of Veterinary Science, Assam Agricultural University, Assam, India

**Keywords:** hemagglutination inhibition, *Lactobacillus reuteri* PIA16, mannan oligosaccharide, Newcastle disease, phytohemagglutinin-P, probiotics

## Abstract

**Background and Aim:**

The chicken gut harbors microflora which impacts the health, production performance and immune response against pathogens. Assam local chickens reared under natural conditions are known to possess high immunocompetence which may be attributable to its gut microbiota make-up. This study aimed to investigate the individual effect of two strains of *Lactobacillus reuteri* PIA16 isolated separately from cecum and jejunum of Assam indigenous chicken on the immunity of broiler chickens against Newcastle disease virus (NDV) when fed singly and in combination with a prebiotic.

**Materials and Methods:**

A total of 240 birds (48 per group) were vaccinated with Lasota strain of NDV on the 5^th^ and 21^st^ day of age. Blood samples were collected before and after immunization against ND for the detection of humoral antibody response by hemagglutination inhibition test. The cell-mediated immune (CMI) response was estimated through response to phytohemagglutinin-P (PHA-P) and expressed as web index.

**Results:**

A significant influence on the immune response to NDV was observed in all the *L. reuteri* PIA16 as well as mannan oligosaccharide (MOS) supplemented groups revealing higher antibody titer than the control counterpart. The CMI response revealed a better cutaneous basophilic hypersensitivity response to PHA-P in the treated groups than the control.

**Conclusion:**

Enhancement in immunity was perceived in the broilers fed with *L*. *reuteri* PIA16 and in combination with MOS due to the stimulation of the host’s humoral and CMI response by the probiotics and prebiotics used.

## Introduction

Newcastle disease (ND) is an infectious and one of the most devastating diseases affecting the poultry which causes very high mortality in chickens and huge productivity losses. To combat diseases, immune status of the chicken is an important factor. The chicken intestine harbors a diverse microflora consisting of both beneficial and pathogenic microorganisms [1]. Colonization of chicken intestine by commensal bacteria begins right after hatch and these bacteria interact closely with cells within the chicken gut-associated lymphoid tissue [2]. This resident microbiota plays a pivotal role in developing the immune system repertoire [3].

The use of probiotics, prebiotics, and symbiotics in poultry nutrition is in vogue as they effectively combat the negative impact of stress or pathogens in poultry. They enrich certain bacterial population in the digestive system which has the potential to reduce chances of infection in poultry and subsequent contamination to poultry products. Probiotics are live microorganisms which, when administered in adequate amounts, confer a health benefit on the host as defined by the FAO/WHO [4]. Probiotics beneficially alter the intestinal microflora balance, inhibit the growth of harmful bacteria, promote good digestion, boost immune function, and increase resistance to infection [5]. Significant enhancement in the immune response was observed in chicken resulting from early colonization of the intestine by probiotic containing *Lactobacillus* *acidophilus* and *Bifidobacterium* *bifidum* [6]. The various species commonly used for probiotic preparations include *Lactobacillus bulgaricus*, *L. acidophilus*, *Lactobacillus casei*, *Lactobacillus helveticus*, *Lactococcus lactis*, *Lactobacillus salivarius*, *Lactobacillus plantarum*, *Streptococcus thermophilus*, *Enterococcus faecium*, *Enterococcus faecalis*, *Bifidobacterium* spp., *Bacillus subtilis*, *Aspergillus oryzae*, *Saccharomyces cerevisiae*, and *Escherichia coli* [7-10]. Recently, potential role for probiotics from fungi as natural growth promoter and effective alternative to antibiotics in broiler production has been reported [11]. Similar mechanism of action of probiotics is seen in prebiotics. Gibson *et al*. [12] defined prebiotic as a selectively fermented ingredient that allows specific changes, both in the composition and/or activity in the gastrointestinal microflora that confers benefits on host well-being and health. Mainly prebiotics are small fragments of carbohydrates and commercially available as oligosaccharides of galactose, fructose, or mannose [13]. Supplementation of either probiotic or prebiotic or both has been reported to improve digestibility and growth performance in broiler chickens [14]. Further, combination of various probiotics when supplemented to layer hens diet increased egg weight, feed efficiency, eggshell quality, decreased cholesterol levels, and increased unsaturated fatty acids in yolk [15].Native breeds of chicken are known for its high immunocompetence, hardiness, better meat quality, desirable taste and flavor of eggs, and meat compared to commercial broilers [16]. Relatively, they have a capacity to resist disease, ability to utilize low-quality feed and their products are preferred by consumers [17]. Assam local chickens reared under natural conditions are also known to possess disease resistance capacity [18].

The gut microflora of Assam local chickens may, therefore, possess certain beneficial microflora to play certain roles in improving the gut immune system. On this context, *Lactobacillus reuteri* PIA16 was isolated from the gut of Assam local chicken for the study and aimed at evaluating the immune response against ND virus (NDV) of the broiler chicken when fed with *L. reuteri* PIA16 singly and in combination with a prebiotic.

## Materials and Methods

### Ethical approval

The study was approved by the Institutional Animal Ethics Committee, Assam Agricultural University, Assam, India.

### Isolation of *Lactobacillus strains*

Two strains of *Lactobacillus* were isolated from the different gut regions of Assam indigenous chicken, i.e., ACE5 (cecum) and AJ3 (jejunum) which were characterized by 16S rRNA gene sequencing and BLAST analysis. Genetic identity of 99.72% with *L. reuteri* was found for both the strains. The identified isolate *L. reuteri* was registered as *L. reuteri* PIA16 under GenBank, National Centre for Biotechnology Information, India.

These strains were treated as two individual entities and were used for *in vivo* growth bioassay where it was fed singly and in combination with a prebiotic. As premix, 20% of daily ration required for broilers was autoclaved and inoculated with 20% of ACE5 and AJ3 broth culture separately and incubated for 37°C for 48 h to facilitate fermentation. Due to readily available source of energy in feed sample, the count of *Lactobacillus* in fermented feed increased to 1.85×10^8^ CFU/g and 1.89×10^8^ CFU/g of fermented feed for ACE5 and AJ3, respectively, which was used in a feeding trial. Mannan oligosaccharide (MOS) was used for the experiment at 0.25% as the prebiotic [19].

A total of 240day-old broiler chicks (Cobb) were randomly distributed into five dietary treatments of eight replicates with six chicks in each replicate reared under cage system for 35 days. The experimental design consisted of T_1_ (Control) – basal diet (mash feed), T_2_ − T_1_+1.85×10^8^ CFU of *L. reuteri* PIA16 (ACE5)/g fermented feed, T_3_ −T_1_+1.89×10^8^ CFU of *L. reuteri* PIA16 (AJ3)/g fermented feed, T_4_ −T_2_+MOS at 0.25%, and T_5_ −T_3_+MOS at 0.25% of feed. Probiotic and prebiotic feeding was from the 1^st^ day of age up to 35 days. The environmental conditions during the experimental period ranged from 26 to 32°C with relative humidity of 65-95%.

### Humoral immune response

The humoral immune response was studied by estimating the ND-hemagglutination inhibition (HI) antibody titers by HI test. The birds were immunized with ND Lasota strain on the 5^th^ and 21^st^ day. Blood samples were collected from 10 broilers per treatment groups for the determination of antibody titer against NDV HI test on day of vaccination and 7^th^, 14^th^, 21^st^, and 28^th^ day of post-vaccination (DPV).

To determine HI titers of the sera samples collected from vaccinated chickens, HI tests were performed using Lasota strain of NDV as per the standard methods described in OIE [20]. Briefly, the 4 HA units of Lasota strain of NDV in equal volume (25 µl) were added to each serum dilution and incubated at 37°C for 45 min. Thereafter, 1% of chicken RBC in 25 µl volume was added to each well and incubated at 37°C for 15 min. The reciprocal of the last serum dilution showing inhibition of hemagglutination of the 4 hemagglutinin units of the NDV was considered as the HI antibody titer of the serum (log_2_ value of HI titer).

### Cell-mediated immune (CMI) response

CMI response was estimated through response to phytohemagglutinin-P (PHA-P) and expressed as a web index. This method was devised by Corrier and De Loach [21]. At 30 days of age, the solution of PHA-P at 0.01 mg/0.05 ml of sterile was injected intradermally in the interdigital space of 3-4 digits of the right foot, and the skin thickness was measured before and after 24 h of injection. The left foot received a similar amount of sterile phosphate-buffered saline (PBS) and served as control, and the difference in skin thickness between injected, and control foot was taken as foot web index. The web swelling of both the feet was measured 24 h after injection by micrometer and the *in vivo* response to PHA-P was expressed as web index.

Foot web index (FWI) was calculated as follows:

CMIR = (R2-R1) - (L2-L1)

Where,

R2 = Thickness after 24 h of PHA-P injection

R1 = Thickness before injection of PHA-P

L2 = Thickness after 24 h of PBS injection

L1 = Thickness before injection of PBS.

### Statistical analysis

The data obtained were statistically analyzed with one-way analysis of variance and Duncan’s multiple range tests to elucidate differences among the treatment groups using the software SAS 9.3 version. Statements of statistical significance were based on p<0.05.

## Results

Significant influence on the immune response to ND virus through dietary supplement of *Lactobacillus* with or without prebiotic was observed. The log2 ND-HI antibody titer did not differ significantly (p>0.05) among different dietary groups till the 5^th^ day of pre-immunization and remained in the range of 2.50±0.17-2.70±0.15. However, the titer started increasing in all the dietary groups, and this trend continued till the 21^st^ DPV and, thereafter, declined (Table-1). Nonetheless, the titer levels of all the *L. reuteri* PIA16 fed groups were found higher than that of the control group. When *L. reuteri* PIA16 was fed along with MOS, the titer level reached to the highest level of 6.40±0.16 as recorded on the 21^st^ DPV in T_5_ group.

**Table 1:** Humoral immune response (log_2_ value of HI titer) to NDV in broiler chickens under different dietary treatments.

Age	Treatment groups				

	T_1_ (Control)	T_2_ (T_1_+ACE5)	T_3_ (T_1_+AJ3)	T_4_ (T_2_+MOS)	T_5_ (T_3_+MOS)
Day old	2.50^a^±0.17	2.60^a^±0.16	2.70^a^±0.15	2.70^a^±0.15	2.70^a^±0.15
5^th^ day	2.10^a^±0.10	2.10^a^±0.10	2.10^a^±0.10	2.10^a^±0.10	2.00^a^±0.00
7^th^ DPV*	4.60^a^±0.16	5.00^ab^±0.21	5.20^b^±0.20	5.40^b^±0.16	5.40^b^±0.16
14^th^ DPV	5.10^a^±0.18	5.50^ab^±0.22	5.70^b^±0.15	5.80^b^±0.13	5.90^b^±0.10
21^st^ DPV	5.60^a^±0.16	6.10^ab^±0.23	6.30^b^±0.21	6.30^b^±0.21	6.40^b^±0.16
28^th^ DPV	5.20^a^±0.13	5.90^b^±0.18	6.00^b^±0.26	6.10^b^±0.18	6.20^b^±0.20

* DPV = Day of post-vaccination;

ab Means bearing same superscripts in a row do not differ significantly (p ≤ 0.05). HI = Hemagglutination inhibition, NDV = Newcastle disease virus, MOS = Mannan oligosaccharide

**Figure-1:**
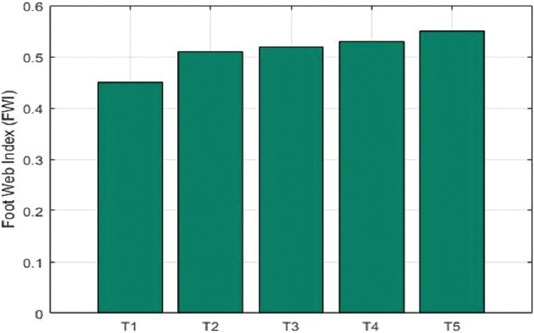
Cell-mediated immune response to phytohemagglutinin-P (mm) in broilers under different dietary treatment.

Results of CMI response through PHA-P stimulation after 24 h are depicted in Figure-1. There was no significant (p>0.05) difference in the cutaneous basophilic hypersensitivity (CBH) response among the five groups, but higher CBH response was observed in all *L. reuteri* PIA16 and MOS-fed groups compared to control at 30 days of age. The mean skin thickness values in response to PHA-P in different dietary groups increased marginally due to *L. reuteri* PIA16 and MOS feeding. However, the increase was insignificant among all the five dietary groups (Figure-1).

## Discussion

The positive effect of feeding *L. reuteri* PIA16 on immune responses was in agreement with other authors [6,22-24] who observed increased antibody titer in chicken post-immunization when fed with probiotic containing *Lactobacillus*. High level of HI titer due to the feeding of probiotic and prebiotic in broilers was reported by several authors [2,25-27]. The primary function of the immune system is to identify and eliminate pathogens [28], and this may be enhanced by administering probiotics that stimulate the local immune system [29]. The most likely reasons for *Lactobacillus* to increase the antibody titer in the present study might be due to the competitive exclusion of pathogens through competition of receptor sites, production of volatile fatty acids that are inhibitory of certain enteric pathogens, productions of bacteriocins or competition with pathogens, and native flora for limiting nutrients or stimulation of a host innate immune response [30].

The present study is in agreement with other authors who reported no significant increase in CBH response to PHA-P while feeding broilers with probiotic, a prebiotic, and acidifier either individually or in combinations [27]. Stringfellow *et al*. [31] and Mahmoud *et al*. [32] observed the lymphocytes from vaccinated broilers treated with probiotic to have greater (*p*<0.05) cell proliferation when compared with the negative control group. Patel [33] reported that the CMI response always exhibited the presence of serological immune response and vice versa and an increase or decrease in the level of CMI response did not always correspond to an increase or decrease in the level of serological immune response. The results of HI and PHA-P tests were independent of each other, and no correlation was found. It was also evident that the cell-mediated immunity played a decisive role in defense mechanisms against ND infection in broilers.

## Conclusion

The *L. reuteri* PIA16 isolated from the gut of Assam indigenous chicken when fed singly and along with MOS to broiler chickens were found to enhance the immunity traits, namely, humoral immunity and CMI response of the broiler chickens. Therefore, supplementation of *L. reuteri* PIA16 along with MOS in commercial broiler chicken ration as probiotic and prebiotics may be proposed for enhancing immunity of the flock, lowering mortality, and boosting the production.

## Authors’ Contributions

DS, GKS, JDM, NK, JKT, and BNS designed the study. GG carried out the research study. PD helped in the study. GG wrote the manuscript. DS, PD, and JDM reviewed and corrected the manuscript. All authors read and approved the final manuscript.
